# Multitarget-Directed Ligands Hitting Serotonin Receptors: A Medicinal Chemistry Survey

**DOI:** 10.3390/ph17091238

**Published:** 2024-09-19

**Authors:** Imane Ghafir El Idrissi, Angela Santo, Enza Lacivita, Marcello Leopoldo

**Affiliations:** Department of Pharmacy-Drug Sciences, University of Bari Aldo Moro, Via Orabona, 4, 70125 Bari, Italy; imane.ghafir@uniba.it (I.G.E.I.); angela.santo@uniba.it (A.S.); marcello.leopoldo@uniba.it (M.L.)

**Keywords:** serotonin, acetyl cholinesterases, butyryl cholinesterases, sigma receptors, multitarget-directed ligands, schizophrenia, depression, Alzheimer’s Disease

## Abstract

Serotonin (5-hydroxytryptamine, 5-HT) is a ubiquitous neurotransmitter in the human body. In the central nervous system, 5-HT affects sleep, pain, mood, appetite, and attention, while in the peripheral nervous system, 5-HT modulates peristalsis, mucus production, and blood vessel dilation. Fourteen membrane receptors mediate 5-HT activity. In agreement with the crucial roles played by 5-HT, many drugs target 5-HT receptors (5-HTRs). Therefore, it is unsurprising that many efforts have been devoted to discovering multitarget-directed ligands (MTDLs) capable of engaging one or more 5-HTRs plus another target phenotypically linked to a particular disease. In this review, we will describe medicinal chemistry efforts in designing MTDLs encompassing activity for one or more 5-HTRs, starting with atypical antipsychotics and moving to dual 5-HT1AR/serotonin transporter ligands, 5-HT6R antagonists/acetyl cholinesterases inhibitors, and 5-HT4R agonists/acetyl cholinesterases inhibitors. We will also provide an outlook on the most recent efforts made in the field.

## 1. Introduction

Drug discovery can proceed through two distinct approaches: target-directed drug discovery (TDD) and phenotypic drug discovery (PDD). While TDD approaches rely on the formulation and testing of specific hypotheses at the molecular level, PDD approaches test compounds in cells or tissues (or even animals) to identify compounds that cause a desirable change in the disease phenotype. The advantages of TDD approaches come from knowledge of the biochemical process underlying the disease, structural biology, and in-silico chemistry-related technologies to provide high-capacity testing of high numbers of compounds and molecular targets. The advantage of PDD approaches is that they test compounds in more realistic systems, e.g., disease-relevant cells, thus gaining results that may be more predictive of the clinical outcome. PDD approaches have considerable challenges, such as hit validation and target deconvolution [1,2].

TDD has been intensely focused on developing molecules designed to act against a specific target with high potency and selectivity. This approach is based on the assumption of a direct cause–effect relationship between the activity of a gene product and a particular phenotype. Consequently, a drug capable of precisely modulating the activity of a deregulated protein could reverse a pathological phenotype. However, the scientific community has recognized that this approach may be too simplistic to address complex multifactorial diseases [3].

On such a basis, the idea of developing drugs that “hit” multiple sensitive nodes belonging to a network of interacting targets, thus offering the potential for higher efficacy and limited drawbacks, gained momentum. As a result, many studies reported multitarget-directed ligands (MTDLs) facing the enormous challenge of ensuring balanced potency and selectivity for the targets, avoiding potentially dangerous off-targets, and satisfying the physicochemical requirements for drug-like molecules [4].

**Figure 1:**
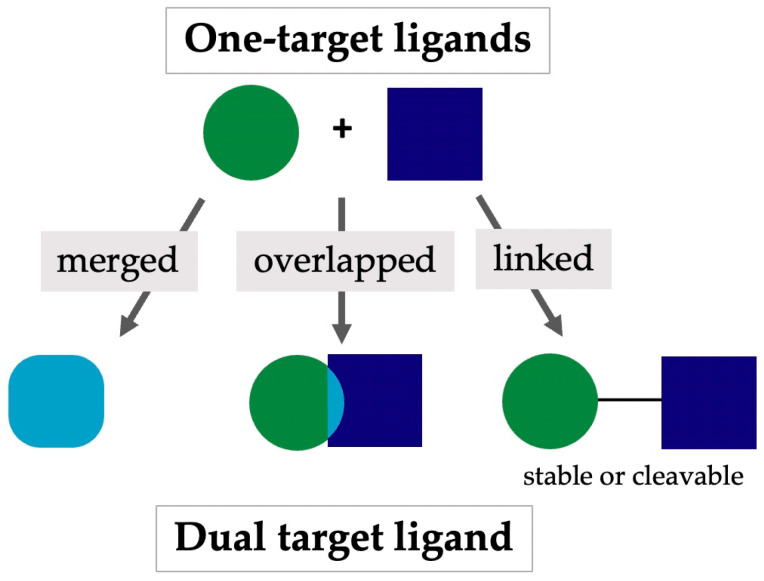
Graphical representation of the design strategies of MTDLs.

The design of MTDLs relies on three widely exploited strategies (Figure 1) that depend on the characteristics of the pharmacophore to employ. The integration of pharmacophores (merge strategy) is applied when two lead compounds share a scaffold structure and some pharmacophoric elements, and the design proceeds by superimposing the two structures and removing the unnecessary groups for the dual interaction. The second strategy involves the overlapping of pharmacophores and is applied when two lead compounds share at least one pharmacophoric element in a structure that features structural elements in distinct locations, ensuring efficient binding to the targets. The third strategy, which involves the linking of pharmacophores, is implemented when there are no similarities between the pharmacophores; therefore, these are simply connected through a linker.

Of these strategies, the merge strategy might produce new chemical entities with better physicochemical properties and higher ligand efficiency than those originating from the other strategies. However, designing merged molecules is complex because the structural requirements to engage the targets properly might be orthogonal [4,5].

Serotonin (5-hydroxytryptamine, 5-HT) is a ubiquitous neurotransmitter in the human body. In the central nervous system, 5-HT affects sleep, pain, mood, appetite, and attention, while in the peripheral nervous system, 5-HT modulates peristalsis, mucus production, and blood vessel dilation [6]. 5-HT activity is mediated by fourteen membrane receptors [6] and dampened down by the serotonin transporter (SERT) that pumps 5-HT released in the synaptic cleft back into neurons [7]. Given the crucial roles played by 5-HT, many established therapeutics target 5-HT receptors (5-HTRs). In addition, SERT inhibitors (selective serotonin reuptake inhibitors, SSRIs) are widely used to treat depression, obsessive-compulsive disorder, and anxiety disorders [8].

Therefore, it is unsurprising that many research groups have designed MTDLs capable of acting on one or more serotonin system-related targets.

**Figure 2:**
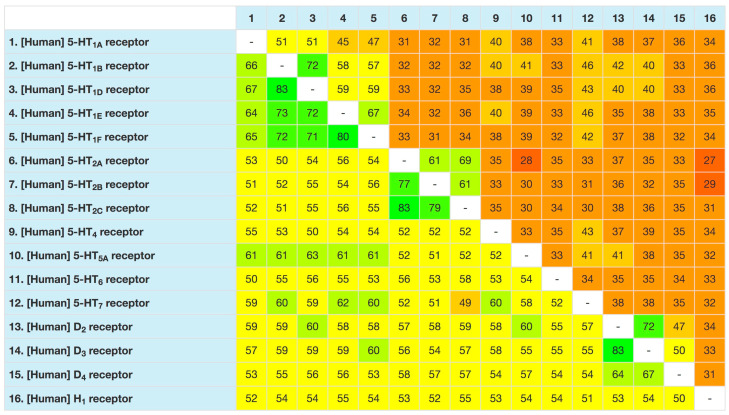
Percentages of similarity (lower-left side) or identity (upper-right side) of selected monoamine receptors. The color code denotes different levels of amino acid sequence identity [17,18].

Several first-generation antipsychotic drugs can be categorized as MTDLs because they exhibit affinity for both the dopamine D2 receptor and a variety of serotonin receptors (Table 1) as the obvious consequence of the high sequence similarity among monoamine receptor proteins (Figure 2). The first step toward the rational combination of dopamine receptor D2 and 5-HT2R antagonism stemmed from the dopamine–serotonin hypothesis of schizophrenia proposed by Herbert Meltzer in 1989 [9]. On this basis, many third-generation atypical antipsychotics were developed, including risperidone, olanzapine, quetiapine, ziprasidone, aripiprazole, lurasidone, cariprazine, and brexpiprazole, most of which display the desired combination of 5-HT2A/D2 receptor antagonism, along with activity for other serotonin receptors that shape the unique profile of each the above antipsychotics [10]. Other combinations of serotonergic and dopaminergic activity have been proposed as a strategy for the treatment of the motor symptoms of Parkinson’s disease by combining dopamine D2/D3 receptor partial agonism with 5-HT1AR agonism [11] or for the treatment of autism spectrum disorder [12].

All the above combinations of activities substantially exploited the integration of pharmacophoric elements (merge strategy) that were mainly overlapping because of the already mentioned high similarity of the primary structure of dopamine and serotonin receptors. Needless to say, most serotonin 5-HT2A, 5-HT1A, and 5-HT7 and dopamine D2, D3, and D4 ligands share the minimal phenylethylamine-resembling pharmacophoric element of protonatable basic nitrogen at a distance of 5.2–5.7 Å from an aromatic ring centroid [13,14]. Therefore, obtaining MTDLs combining serotonin and dopamine receptor activity relies on subtle modifications of 1-arylpiperazine- or 4-arylpiperidine-containing molecules to achieve the desired combination of activity. This review will not cover such types of structural explorations, focusing instead on the less immediate exploitation of MTDL design. Readers interested in MTDLs combining affinity for multiple serotonin and dopamine receptors can refer to some recently published reviews [10,15,16].

## 2. Multitarget-Directed Ligands Encompassing Serotonergic Activity

### 2.1. Multitarget-Directed Ligands Targeting Serotonin Receptors and Serotonin Transporter

Selective serotonin reuptake inhibitors (SSRIs) are the most prescribed drugs to treat major depressive disorder. Yet, SSRIs present a slow onset of the clinical effect, at least in part, due to the activation of a negative feedback mechanism, primarily through 5-HT1A autoreceptors [19]. Toward the aim of obtaining rapid-acting antidepressants, various studies have pointed to the combination of SERT inhibitory activity with modulation of 5-HT1AR. Preclinical studies showed that co-administration of an SSRI with a 5-HT1AR antagonist like pindolol or WAY 100,635 resulted in a faster and/or enhanced antidepressant effect [20,21]. This effect was related to avoiding 5-HT1A autoreceptor desensitization, which in turn causes an increased baseline firing rate of serotonergic neurons. On the other side, co-administration of an SSRI with a 5-HT1AR agonist/partial agonist produces a rapid increase in serotonergic system neurotransmission via stimulation of postsynaptic 5-HT1ARs [22].

Starting from 1998, more than fifty original medicinal chemistry studies have been published on the development of dual 5-HT1A/SERT ligands. Here, we will retrace the efforts of researchers from both industry and academia.

**Figure 3:**
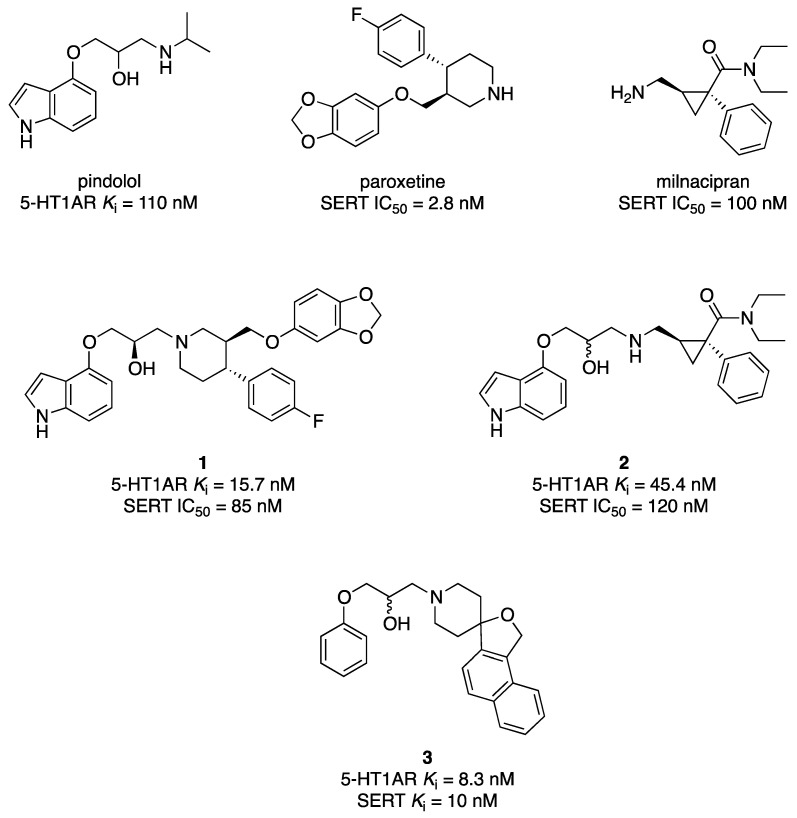
Structural formulas of SSRIs and dual 5-HT1AR/SERT ligands developed by Pierre Fabre and Merck, Sharp & Dohme [23,24].

In 1998, researchers at Pierre Fabre reported on the design of hybrid molecules that featured structural characteristics of the 5-HT1AR antagonist pindolol and SERT inhibitors, having a basic nitrogen as common structural element [23]. Through an overlapping approach, they designed hybrids inspired by norfluoxetine, milnacipram, and paroxetine, eventually finding two hybrids (compounds **1** and **2**) that combined 5-HT1AR affinity and SERT inhibitory activity (Figure 3). These compounds showed higher 5-HT1AR affinity than pindolol and SERT inhibitory activity comparable to the starting SSRIs.

Pindolol was the starting point for developing dual 5-HT1AR/SERT ligands in a study performed by researchers at Merck, Sharp & Dohme. In this study, a spirofused piperidine with inhibitory SERT activity was incorporated into a set on pindolol-inspired structures [24]. The study identified compound **3** as a balanced dual 5-HT1AR/SERT ligand, with excellent pharmacokinetic properties (Figure 3).

**Figure 4:**
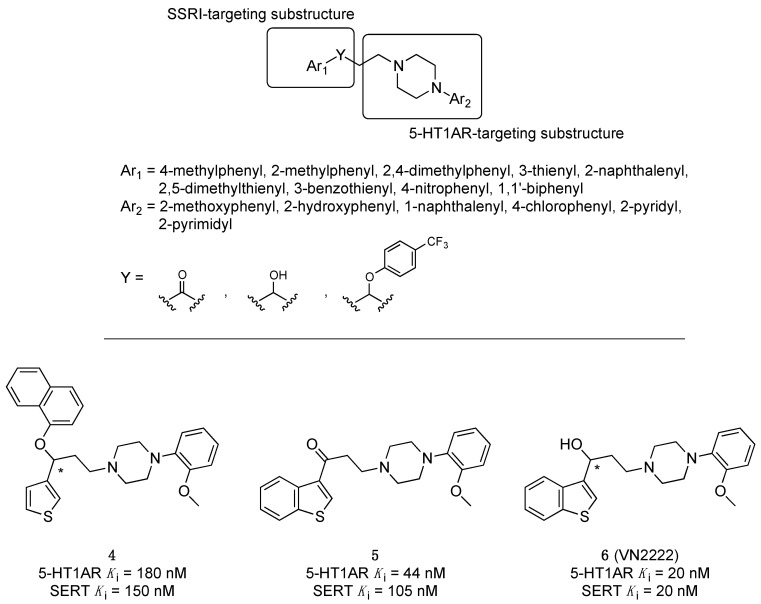
Structural formulas of dual 5-HT1AR/SERT ligands developed by Monge’s group. The asterisk indicates that the compounds were tested as racemates [25,26].

Monge’s group pursued the strategy of overlapping a structure capable of binding 5-HT1ARs and SERT [25,26], starting from a γ-phenoxypropylamine moiety (with SERT inhibiting properties) and arylpiperazine structures, as long-chain arylpiperazines represent one of the most thoroughly studied class of 5-HT1AR ligands (Figure 4). The SARs evidenced that (i) introducing an aryl ether moiety at Y was unnecessary to obtain dual 5-HT1AR/SSRI activity. The best results were obtained when Y = CHOH; (ii) the highest affinities were obtained when Ar2 was 2-methoxyphenyl; (iii) concerning Ar2, affinities increased when the benzene ring was replaced by thiophene and, in the series of benzocondensed derivatives, when naphthalene was replaced by benzothiophene. The authors highlighted compounds **4**, **5**, and, especially, **6**, also known as VN2222, with balanced affinity at both 5-HT1ARs and SERT and efficacy in animal models of depression (Figure 4). VN2222, the most representative compound of the series, was also endowed with high affinity at other monoamine receptors expect for α1-adrenoceptors. VN2222 inhibited 5-HT uptake both in vitro (rat cortical synaptosomes, mesencephalic cultures) and in vivo (local administration into the rat ventral hippocampus) and behaved as a 5-HT1AR partial agonist. VN2222 had remarkable activity in a predictive rat model of depression (the learned helplessness test) [27].

**Figure 5:**
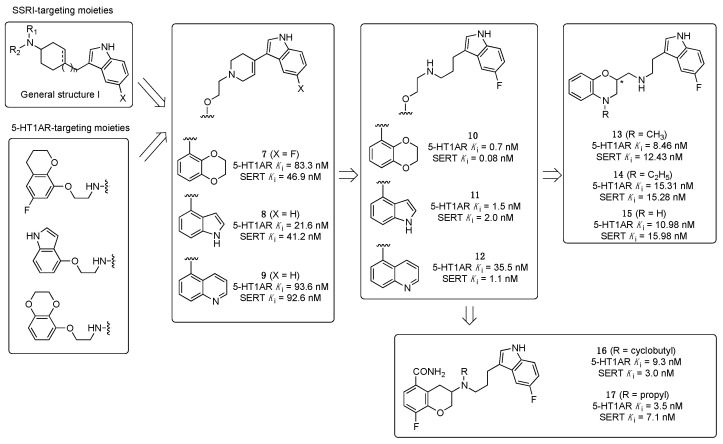
Design strategy pursued by Wyeth-Ayerst Laboratories to identify dual 5-HT1AR/SERT ligands. The asterisk indicates that the compounds were tested as racemates [28,29,30,31].

For almost a decade, researchers at Wyeth-Ayerst Laboratories made several studies to identify dual 5-HT1AR/SERT ligands. Their starting design concept was to modify cyclohexyl indoles exemplified by General Structure I, known as SERT ligands, by systematically introducing the 5-HT1AR pharmacophoric requirements (Figure 5).

To this end, aryloxyethylamines were selected as 5-HT1AR pharmacophores and were merged with the cyclohexyl indoles by inserting the basic nitrogen in the cyclohexyl ring (compounds **7**–**9**, Figure 5). The compounds showed a balanced affinity for 5-HT1ARs and SERT and, importantly, acted as 5-HT1AR antagonists [28]. To improve the potency at both targets, more flexible secondary amines were investigated by opening the piperidine ring, leading to compounds **10**–**12** (Figure 5), which showed activity in the nanomolar or subnanomolar range [29]. However, the main shortcoming observed in this series was the lack of selectivity over adrenergic α_1_ receptor. Therefore, the aryloxyethylamine moiety was constrained within the benzoxazine structure. Although this structural modification did not significantly affect the affinity for 5-HT1ARs and SERT, the compounds were found to act as 5-HT1AR agonists and, thus, were not developed further (compounds **13**–**15**, Figure 5) [30]. In another series, the aryloxyethylamine moiety was constrained in a 3-amino-3,4-dihydro-2*H*-1-benzopyran nucleus, inspired by the 5-HT1AR antagonist robalzotan (compounds **16** and **17**, Figure 5). Among the studied compounds, derivative **16** showed good 5-HT1AR and SERT binding affinities, full antagonism at 5-HT1ARs, and greater than 100-fold selectivity over other aminergic receptors. When tested in vivo, compound **16** acutely elevated serotonin levels in the rat frontal cortex to a similar extent to chronic (14-day) SSRI treatment [31].

**Figure 6:**
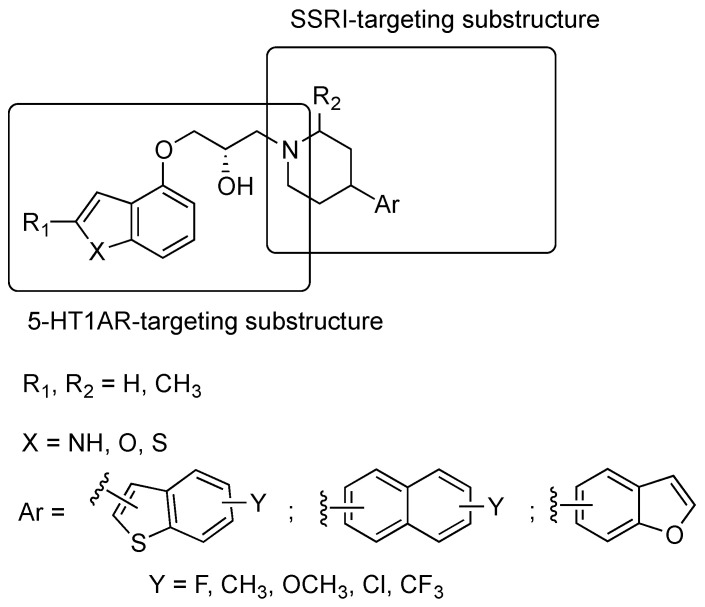
Graphical summary of the structures of dual 5-HT1AR/SERT ligands studied by Eli Lilly [32].

From 2003 to 2006, researchers at Eli Lilly reported on discovering potent dual 5-HT1A/SSRI compounds. Their research identified many compounds showing high affinity for 5-HT1AR and SERT in vitro (Figure 6). The extensive SAR exploration led to identifying the 4-(benzothiazol-2yl)piperidine derivatives characterized by high affinities for the targets, as exemplified by compound **18** (Table 2). However, ex vivo evaluation of the binding to 5-HT1ARs and SERT evidenced serious limitations in actually binding both targets in vivo. Refinement of the structure, focusing on the disubstituted piperidine ring stereochemistry, led to compound **22** (Table 2) with balanced ex vivo occupancy of 5-HT1ARs and SERT. The best compound of the whole series was derivative **24** (Table 2), as it confirmed the desired blockade of 5-HT1ARs and inhibition of SERT in vivo. Compound **24** had no negative feedback effect on the 5-HT neuronal activity elicited by fluoxetine alone via activation of somatodendritic 5-HT1A autoreceptors [32].

A milestone in this field was the FDA approval in 2011 of the mixed SSRI and 5-HT1AR partial agonist vilazodone for the treatment of major depressive disorder in adults [33]. A detailed study reporting how vilazodone was discovered had been published seven years earlier [34].

**Figure 7:**
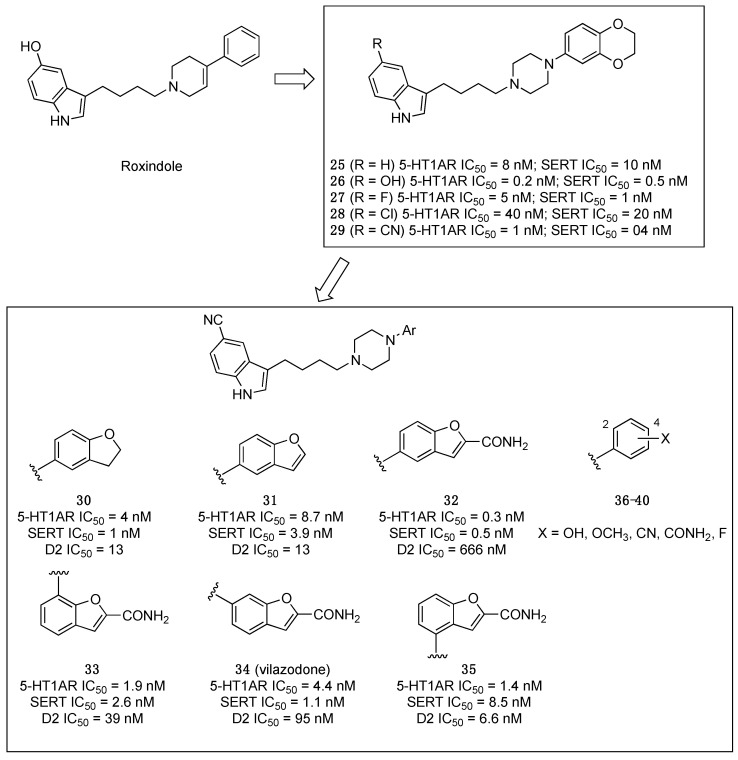
Graphical summary of the studies that led to the discovery of vilazodone by Merck, Sharp & Dohme [33,34].

The starting point was roxindole and related arylpiperazine derivatives, which were characterized as potent 5-HT1A ligands featuring affinity for SERT. Thus, the authors, who had already established the SAR for 5-HT1ARs with these arylpiperazine derivatives, explored the SAR of the arylpiperazines, exemplified by compounds **25**–**29** (Figure 7), intending to improve SERT affinity and, most importantly, reducing dopamine D2 receptor affinity. The best substituents on the indole ring were –F and –CN. Therefore, the subsequent modifications were performed on 5-fluoro- or 5-ciano-substituted indole derivatives. The following modification targeted the aryl ring linked to the piperazine nitrogen to reduce D2 receptor affinity. Various aryl rings were investigated, including substituted phenyl rings that were still high-affinity ligands for the D2 receptors. Yet, various condensed bicyclic derivatives, including the benzofuran ring, were considered as substituents. Investigation of the optimal connection position led to vilazodone (compound **34**, Figure 7).

**Figure 8:**
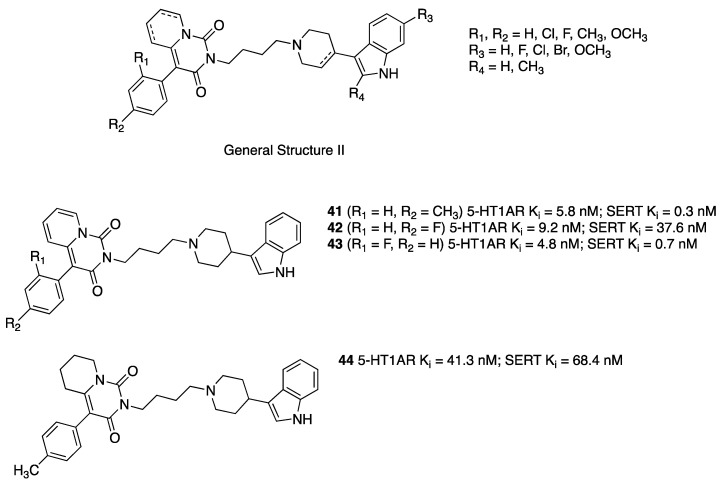
Structural formulas of 4-(1*H*-indol-3-yl)-1-piperidyl-based dual 5-HT1AR/SERT ligands [35].

Starting from 2009, researchers from various academic institutions in Poland described multiple dual 5-HT1A/SERT ligands. They initially combined the terminal fragment of certain long-chain arylpiperazine derivatives previously studied as 5-HT1AR ligands with the 3-(4-piperidyl)-1*H*-indole group known for inhibiting SERT by designing about 80 new compounds belonging to General Formula II (Figure 8) that showed high to moderate binding affinity to 5-HT1ARs and SERT. The most representative compounds were **41**–**43** (Figure 8) [35,36]. In vivo tests showed that **41** and **42** behaved as 5-HT1AR agonist pre- and postsynaptically at 10 mg/kg and 5 mg/kg doses (i.p.), respectively.

**Figure 9:**
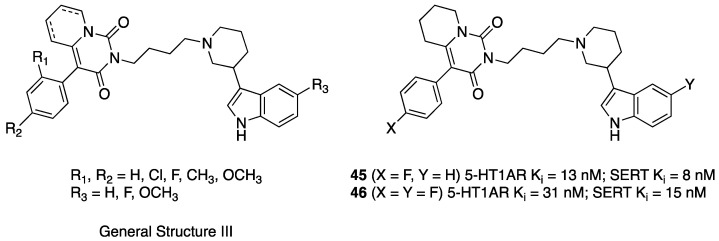
Structural formulas of 3-(1*H*-indol-3-yl)-1-piperidyl-based dual 5-HT1AR/SERT ligands [36].

The same authors investigated analogs of compounds **41**–**43** and **44**, featuring a 3-(1*H*-indol-3-yl)-1-piperidyl-, instead of the 4-(1*H*-indol-3-yl)-1-piperidyl moiety (General Structure III, Figure 9) for a total 27 pairs of pyrido[1,2-c]pyrimidine/5,6,7,8-tetrahydropyrido[1,2-c]pyrimidine derivatives. In general, 5,6,7,8-tetrahydropyrido[1,2-c]pyrimidine derivatives showed higher affinity for both 5-HT1ARs and SERT when compared with the pyrido[1,2-c]pyrimidine counterpart. The affinity range of 5,6,7,8-tetrahydropyrido[1,2-c]pyrimidine derivatives was Ki = 8–259 nM for 5-HT1ARs and Ki = 8–602 nM for SERT. These compounds, however, displayed moderate to low in vitro metabolic stability. The most representative compounds **45** and **46** had the properties of presynaptic antagonists at 5-HT1ARs in in vivo tests in mice.

**Figure 10:**
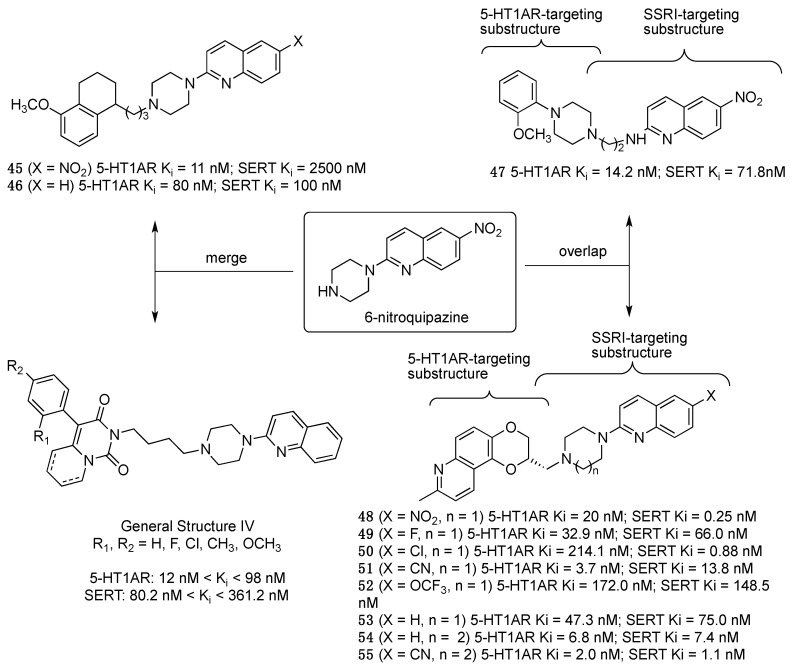
Design strategies of dual 5-HT1AR/SERT ligands starting from 6-nitroquipazine [38,39,40].

6-Nitroquipazine, a potent and selective inhibitor of the synaptosomal uptake of 5-HT with 1000-fold selectivity over NET and DAT [37], was the focus for developing dual 5-HT1AR/SERT inhibitors. The first attempt incorporated the 6-nitroquipazine structure into the general formula of long-chain arylpiperazine, a scaffold widely exploited to find 5-HT1AR ligands. Perrone et al. exploited both the merge and overlap strategies for incorporating the 6-nitroquipazine scaffold (structures A and B, Figure 10) [38]. Compound **47** (Figure 10) showed balanced affinity for 5-HT1ARs and SERT, agonist activity at 5-HT1ARs, and the ability to inhibit SERT activity in rat synaptosomes.

Using a similar approach, Gomołka et al. merged the quipazine nucleus with different 4-aryl-pyrido[1,2-c]pyrimidines, obtaining a series of compounds characterized by a wide range of affinity for 5-HT1ARs and SERT (General Structure IV, Figure 10) [39]. Using the overlap approach, Wyeth reported a series of quipazine derivatives (compounds **48**–**55**, Figure 10) characterized by a nanomolar affinity for 5-HT1ARs and SERT. Compound **51** was further profiled in vivo and was able to increase the 5-HT level in a dose-dependent manner after oral administration in a way consistent with a more rapid antidepressant-like effect [40]. Based on in vitro and in vivo evidence showing that chronic administration of SSRIs desensitizes 5-HT1B autoreceptors [41], it was proposed that, in addition to targeting 5-HT1ARs, the function of 5-HT1BRs may be targeted to obtain fast-acting antidepressants. This hypothesis was corroborated by studies showing that co-administration of 5-HT1AR and 5-HT1BR (or 5-HT1B/DR) antagonists can result in an additive increase in SSRI effects [42]. Therefore, a compound incorporating SERT inhibitor and 5-HT1A/1B autoreceptor antagonist properties could be effective as a rapid-onset antidepressant.

Researchers at Glaxo Smith Kline, starting from screening hit **56**, featuring high 5-HT1AR affinity and moderate SERT potency, undertook a medicinal chemistry campaign aimed at reducing the affinity for the off-target adrenergic beta-2 receptor.

**Figure 11:**
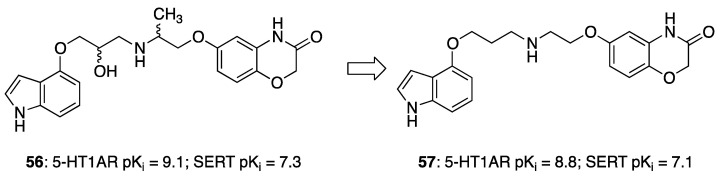
Starting point of the medicinal chemistry campaign to identify mixed 5-HT1/SERT ligands [43].

The first step was removing the hydroxy group from the linker to reduce beta 2 affinity (compound **57**, Figure 11). Next, the effect of the increased conformational constraint of the central linker was investigated to increase the affinity for the target proteins, leading to the piperidinyloxy analog **58** (Table 3). Next, SAR exploration on the left-hand aryloxy group probed with about 90 variations indicated that bicyclic aryl groups had the preferred profiles. The encouraging profile of 1-naphthyl derivative **59** (Table 3) led to the investigation of the isoquinolinyl and quinolinyl derivatives **60**–**63**. The 5-quinolinyloxy derivative **63** featured the lowest intrinsic activity at 5-HT1ARs. This finding prompted the authors to replace the piperidinyloxy linker of compound **63** with a range of alternative basic linkers. The best results were obtained with compound **66**, which was metabolically unstable because it was metabolized by aldehyde oxidase at C-2 of the quinoline ring. The introduction of a methyl substituent at C-2 led to identifying compound **67** (SB-649915, Table 4) as a high-affinity 5-HT1AR antagonist with potent SERT inhibitory activity. Further characterization of this compound revealed significant 5-HT1B/1DR antagonist activity, providing Glaxo Smith Kline researchers the tool for probing the abovementioned hypothesis. Of note SB-649915 had a favorable ADME profile in rats [43]. A subsequent study focused on modifying the central 2-ethoxypiperidine linker in SB-649915 to assess the correlation between the potency at SERT and 5-HT1A/1B/1DRs. To this end, the authors explored the possibility of engaging pockets where the residues were conserved across these subtypes besides the conserved aspartate on TM3. Thus, they designed a set of compounds with various alternative linkers to probe the binding pockets and ensure high affinity at 5-HT1A/1B/1DRs and SERT (compounds **68**–**75**, Table 4) [44].

An additional target investigated to confer a faster onset of action for SSRIs is 5-HT2CR, for which antagonism may improve certain co-morbid symptoms of depression (anxiety, perturbed sleep, sexual dysfunction) [45].

**Figure 12:**
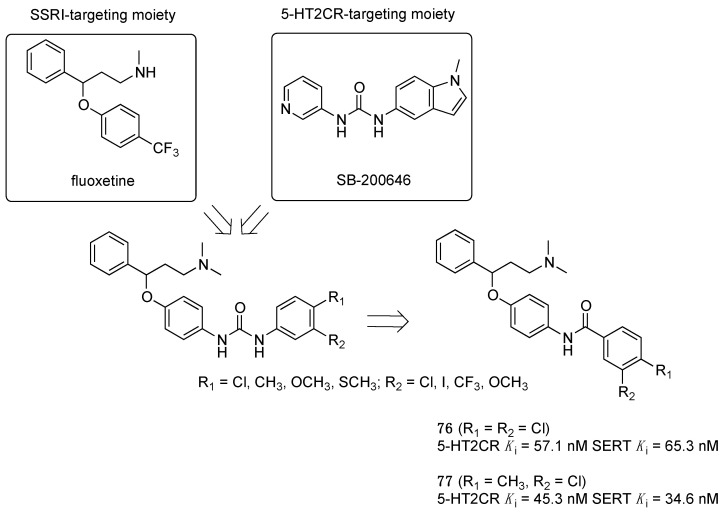
Design of dual 5-HT2CR/SERT ligands [46].

Researchers at Gedeon Richter Plc. applied a knowledge-based pharmacophore hybridization approach to design compounds possessing dual 5-HT2CR/SERT inhibitory activity, starting from fluoxetine that also binds 5-HT2CRs, in addition to SERT, suggesting some overlap of the two pharmacophores. As a 5-HT2CR-privileged motif, the biaryl urea scaffold present in various 5-HT2CR antagonists was selected (SB-200646). The hybridization strategy identified a few phenylurea and benzamide derivatives with dual 5-HT2CR/SERT inhibitory activity. Although the study identified the two well-balanced compounds **76** and **77** (Figure 12), both having good metabolic stability and acceptable penetrability, no subsequent studies were published to show the antidepressant activity of these compounds [46].

Independent studies have described how the combined administration of low doses (that are ineffective by themselves) of an SSRI and the selective 5-HT7R antagonist SB 269,970 induces an antidepressant response in behavioral models [47,48].

**Figure 13:**
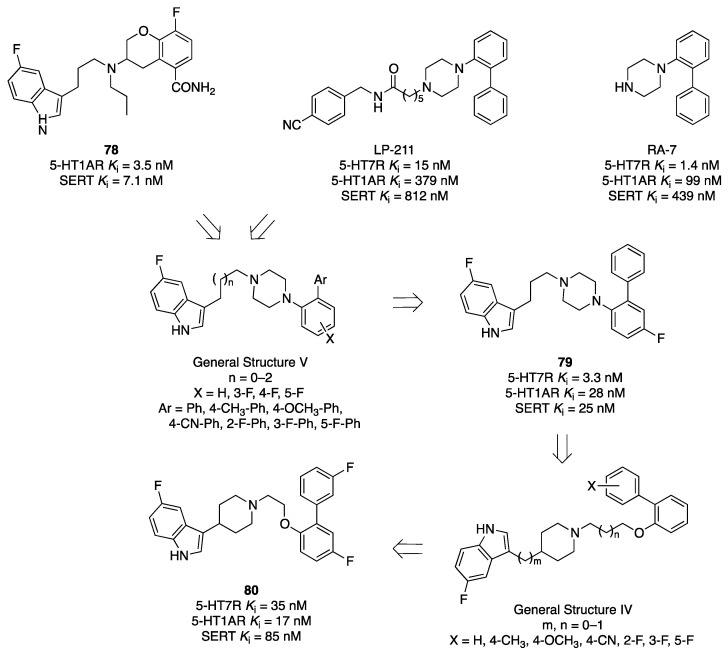
Design and evolution of 5-HT1A/5-HT7R/SERT ligands [49,50].

Such observations prompted a group from the Shanghai Institute of Pharmaceutical Industry to pursue the objective of developing novel antidepressants combining dual 5-HT1A/SERT affinity with 5-HT7R inhibition. The authors took advantage of privileged frameworks already exploited for developing 5-HT1AR/5-HT7R ligands and SERT inhibition properties (Figure 13). In particular, to enhance affinity for 5-HT1ARs and 5-HT7Rs, the 1-biphenylpiperazine scaffold of the selective 5-HT7R agonist LP-211 and its analog RA-7 was chosen. Of note, both compounds presented an affinity for SERT [49]. For SERT affinity, the authors selected the 5-fluoro-3-indolyl moiety present in several dual 5-HT1AR/SERT ligands developed by Wyeth (see compound **7** in Figure 5). Through systematic investigation of the optimal length of the alkyl chain between the 5-fluoro-3-indolyl and the piperazine ring and decoration of the biphenyl system with one or more substituents, the authors ended up optimizing compound **79** that showed balanced affinity for the target proteins, good oral pharmacokinetic properties in rats, and an acceptable hERG profile. Compound **79** was further evaluated using the mouse forced swimming test and tail suspension test (oral administration once daily for seven days at doses of 10, 20, and 40 mg/kg/day), showing a dose-dependent reduction of the immobility time in the forced swimming test and the immobility time in tail suspension test. In these tests, compound **79** showed similar efficacy to the reference antidepressants vortioxetine and venlafaxine [50]. In a follow-up paper, the same research group investigated the linker replacement between the 5-fluoro-3-indolyl and biphenyl moieties of compound **79**, following a similar strategy implemented by Glaxo Smith Kline researchers ten years earlier (see ref. [44]). The study exploited the 3-(4-piperidyl)-1*H*-indole scaffold, already known for inhibiting SERT. The best compound of the series was **80**, which was less potent than **79** in the forced swimming test and tail suspension test in the mouse (oral administration once daily for seven days at doses of 10, 20, and 40 mg/kg/day) [51]. These studies do not provide insights into the advantages offered by targeting SSRI/5-HT1A/5-HT7 in terms of therapeutic outcomes concerning established antidepressants like vortioxetine or venlafaxine.

### 2.2. Multitarget-Directed Ligands Targeting Serotonin Receptors and Norepinephrine Transporter

**Figure 14:**
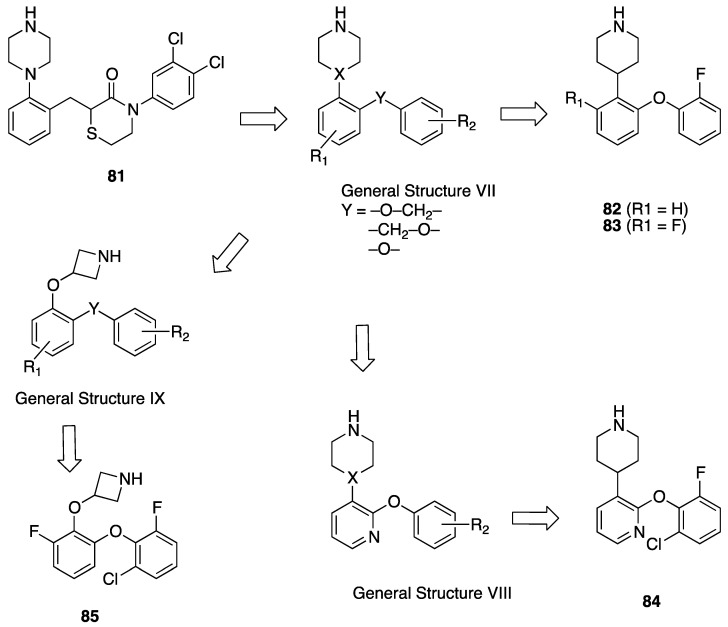
Design and evolution of 5-HT1A/NET ligands [54,55].

Preclinical behavioral studies suggested that the antidepressant efficacy of *N-*desalkylquetiapine (an active metabolite of quetiapine) was mediated through selective norepinephrine (NE) reuptake inhibition and 5-HT1A and 5-HT7 receptor activities [52]. On this basis, researchers at Pfizer worked to identify compounds featuring dual norepinephrine reuptake inhibitory (NRI) activity and 5-HT1AR agonism by initially screening the corporate library. They found the aryl piperazine thiomorpholinone **81** (Figure 14) as a promising dual-activity molecule. Guided by computational analysis in an empirical NRI pharmacophore model, the authors concluded that the size of compound **81** might be unnecessarily fairly large. Thus, several truncated analogs were designed, characterized by General Formula VII (Figure 14). Evaluation of various ether linkage led to diphenyl ethers displaying NET and 5-HT1AR binding affinity, accompanied by meaningful selectivity over SERT and DAT. In particular, piperidine diphenyl ether derivatives were potent and selective for the desired targets. Among these, the 2-fluoro substituted compounds **82** and **83** emerged as preferable because they combined 5-HT1AR partial agonism and functional NET inhibition with selectivity versus DAT and SERT. Both compounds were also characterized by low microsomal clearance [53].

The optimization was pursued further by investigating alternative templates in which one of the phenyl rings was replaced with a heterocyclic system (General Formula VIII, Figure 14). The key results of the SAR studies were that (i) the piperidine ring was preferred over the piperazine ring because the former retained higher NET inhibitory activity, most likely due to higher basicity, compared to piperazine; (ii) phenyl allows substituents in the ortho position relative to the oxygen bridge, whereas small substitutions are allowed in the para position. The best compound of the series was **84**, which showed the desired profile at NET and 5-HT1ARs. In addition, ex vivo occupancy studies demonstrated that **84** was brain penetrant and could bind to the target proteins in rats in vivo (10 mg/kg s.c.). Ex vivo NET and 5-HT1AR occupancies (75.3% for NET and 37.4% for 5-HT1AR agonist) were consistent with values required to elicit functional activity in vivo [54].

The final step of Pfizer’s campaign on this topic regarded the replacement of the piperidine ring in the structures with General Formula VIII by an azetidine with a one-atom linker (oxygen), as it would preserve the distance between the basic nitrogen and the central phenyl ring (General Formula IX, Figure 14). This modification led to compounds with the desired profile, exemplified by compound **85**, which displayed the desired profile at NET and 5-HT1ARs, very good ex vivo occupancy of the target proteins, no activity at SET and DAT, and good oral exposure in dogs [55]. Pfizer did not develop these compounds further.

### 2.3. Multitarget-Directed Ligands Targeting Serotonin Receptors and Sigma Receptors

The “sigma enigma” has fascinated researchers since the mid 1970s, when radioligand binding experiments evidenced that the sigma receptor binding site was distinct from the opioid receptors. The unusual pharmacological profile of the sigma receptor and its promiscuous ligand binding profile complicated the efforts to ascribe pharmacological effects to it unambiguously. The discovery of [^3^H](+)-pentazocine enabled the identification of two distinct sigma receptors: the sigma 1 receptor that broadly corresponds to the initially defined sigma receptor and the sigma 2 receptor characterized by high affinity for ditolylguanidine (DTG) and haloperidol [56]. The sigma 2 receptor is now identified as transmembrane protein 97 (TMEM97) [56].

The sigma 1 receptor is present throughout the CNS. It has been implicated in various neurological diseases, such as analgesia, anxiety, depression, drug addiction, learning, memory deficit disorders, motor disorders, schizophrenia, and psychosis [57].

The sigma 2 receptor has attracted considerable interest as a therapeutic target for the treatment of breast cancer, lung cancer, melanoma, ovarian cancer, pancreatic cancer, and prostate cancer. Sigma 2 receptors overexpressed in tumor cells can be the target for therapies as they mediate many cellular responses unique to tumor cells [58]. The sigma 2 receptor is also involved in neurological diseases, such as age-related degenerative diseases of the central nervous system, including Alzheimer’s disease, α-synucleinopathies, and dry age-related macular degeneration [59].

Early studies showed that many drugs that bind 5-HTRs could also bind the sigma receptor binding site, thus highlighting a high degree of overlap between the structural requirements for interacting with 5-HT1A or 5-HT2A and sigma receptors (Table 1).

**Figure 15:**
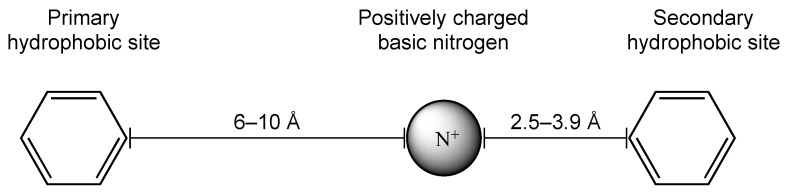
The sigma 1 receptor pharmacophore model (adapted from ref. [60]).

In 1994, Glennon proposed a pharmacophore model of the sigma 1 receptor that included one positively charged nitrogen flanked by two aromatic or hydrophobic moieties placed at optimal reciprocal distances (Figure 15) [60].

High affinity was obtained with 2-phenylethyl amine derivatives, a structural framework also embedded in 5-HT1AR and 5-HT2AR ligands. Therefore, early studies pursued sigma 1-selective ligands, treating 5-HTRs as off-target receptors (see as an example refs. [61,62,63]).

Yet, the 1-arylpiperazine derivative OPC-14523, a sigma and 5-HT1A receptor agonist, showed antidepressant-like effects in animal models of behavioral despair. Both mechanisms contribute to an acute antidepressant-like effect that is not related to SERT inhibition nor monoamine oxidase inhibition [64].

In addition, recent studies have suggested that combined activation of the 5-HT1A and sigma 1 receptors by fluvoxamine has an anti-anhedonic effect associated with the activation of the prefrontal dopaminergic system [65]. Therefore, the deliberate design of dual 5-HT1AR/sigma 1 agonists endowed with suitable drug-like properties could be further exploited. From a medicinal chemist perspective, the design of 5-HT1AR ligands featuring sigma activity is an involuntary application of the overlapping strategy due to the close resemblance of the pharmacophores. In this respect, valuable examples include the 4-arylpiperazine/4-arylpiperidine derivatives reported by Perregaard and coworkers, showing an affinity for sigma 1/sigma 2/5-HT1A/5-HT2A receptors (compounds **86**–**90**, Table 5) [66].

More recently, Porter et al. described some tetrahydroquinolines and 3-amino-chromanes embedding the phenethylamine backbone with affinity for a subset of 5-HTRs and sigma receptors. The study aimed to find selective ligands, yet it provides clues on generating new multitarget-directed ligands encompassing sigma and serotonergic activity (compounds **91**–**93**, Table 6) [67].

**Figure 16:**
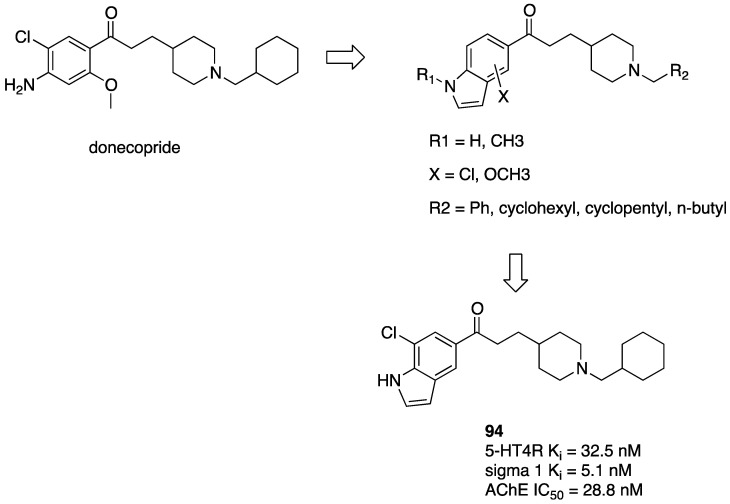
Design of MTDLs originating from donecopride with activity at 5-HT4Rs, sigma 1 receptors and AChE [68].

The search for sigma 1/5-HT4R ligands originated from discovering that donecopride, a 5-HT4R partial agonist with AChE inhibitor activity (see Section 2.4), also had a high affinity for the sigma 1 receptor (Figure 16). This notion inspired modifications to find compounds featuring such combinations of activities. The benzene ring of donecopride was replaced by a variously decorated indole ring, and donecopride’s basic nitrogen was substituted with various groups, including benzyl cyclopropyl and *n*-butyl. Compound **94** showed a balanced potency toward the three targets [68].

### 2.4. Multitarget-Directed Ligands Targeting Serotonin Receptors and Acetyl Cholinesterase/Butirryl Cholinesterase

**Figure 17:**
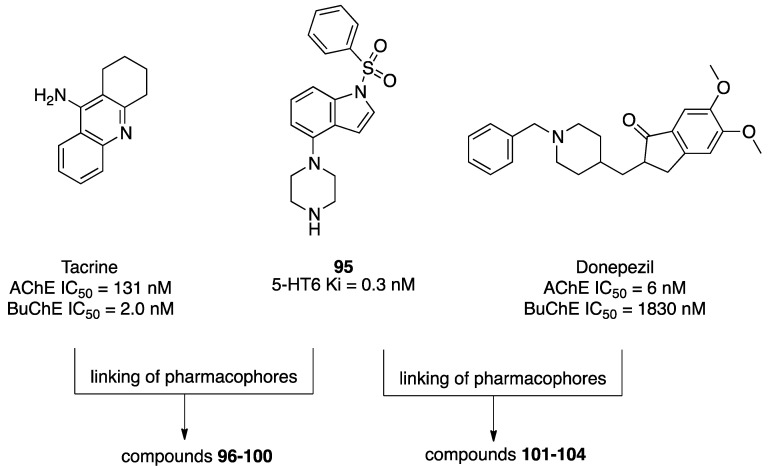
Design strategy of MTDLs targeting 5-HT6R, AChE, and BChE [71,72,73].

A growing body of evidence suggests that brain 5-HT circuitry plays an important role in the development of Alzheimer’s disease (AD) and related cognitive and behavioral impairments. In addition to its role in regulating proliferation, differentiation, maturation, and programmed death of neurons, 5-HT is also involved in forming insoluble aggregates of β-amyloid and tau protein [69]. Several 5-HTRs and the intracellular pathways activated by them have a role in the pathological processes of the disease [69] and, as such, can be exploited to develop potential drugs for AD in combination with activities towards other targets. Więckowska and colleagues have developed MTDLs that modulate the function of 5-HT6R, acetylcholinesterase (AChE), and butyrylcholinesterase (BChE). Among the 5-HTRs, 5-HT6Rs are almost exclusively distributed in the brain areas involved in learning and memory processes, including the hippocampus and cortex, and the blockade of the receptor has procognitive effects in several animal models. Interestingly, 5-HT6R blockade indirectly triggers acetylcholine release [70], contributing to the procognitive effects and inhibiting the formation of β-amyloid aggregates [69]. The design of MTDLs started from the 5-HT6R antagonist 1-(phenylsulfonyl)-4-(piperazin-1-yl)-1*H*-indole (compound **95**, Figure 17) that was linked to tacrine and donepezil for AChE inhibition and phthalimide for BChE inhibition using alkyl linkers of different lengths (Figure 17) [71,72,73].

The biological data evidenced that the conjugation of compound **95** with the AChE/BChE pharmacophore did not affect the affinity for 5HT6R dramatically (Table 7). This was likely due to the retention of the crucial interactions for 5-HT6R represented by the charge-reinforced hydrogen bond between the protonated piperazine nitrogen and the carboxyl group of Asp3.32, a conserved amino acid in all monoaminergic receptors, and by the aromatic (CH-π) interactions established by the indole moiety and the phenyl ring with hydrophobic amino acid residues into the binding site. Surprisingly, the tacrine derivatives **96**–**99** (Table 7) were more potent at AChE than tacrine itself (AChE IC_50_ = 131 nM, hBChE IC_50_ = 2.0 nM), most likely because of the formation of additional interactions by the arylpiperazine fragment within the AChE catalytic site. Replacing the tacrine fragment with benzylamine (compounds **101** and **102**, Table 7) was detrimental to AChE and BChE activity. The authors hypothesized that the higher basicity of *N-*benzylamine fragment compared to *N-*benzylpiperidine of donepezil (calculated pKa = 9.67 vs. 8.54) drove the compounds deeper into the catalytic site, thus preventing the formation of favorable aromatic interactions in the peripheral site. The phthalimide fragment should provide activity at BChE, but compounds **104** and **104** (Table 7) did not show activity for either of the target enzymes. As for the alkyl linkers, two distinctive binding modes were predicted for short-chain compounds (e.g., compound **96**) and long-chain compounds (e.g., compound **97**). In both compounds, the 1,2,3,4-tetrahydroacridin-9-amine fragment was able to reach the catalytic site of AChE; however, for the short-chain compounds, the tacrine fragment assumed a perpendicular position in the catalytic site as compared to the long-chain compounds, thus providing a slightly lower potency.

**Figure 18:**
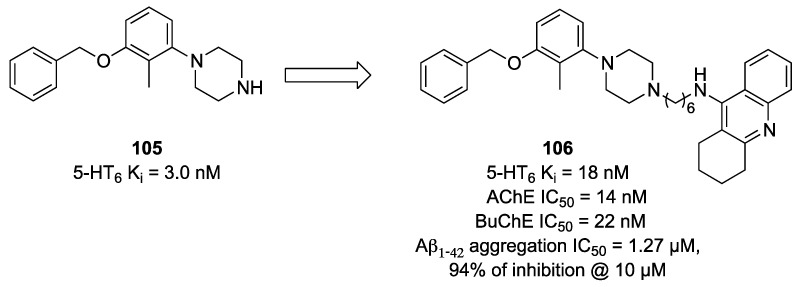
Piperazine-based MTDLs targeting 5-HT6R, AChE, and BChE [72].

In a subsequent study, 1-[3-(benzyloxy)-2-methylphenyl]piperazine (compound **105**, Figure 18) was selected as the 5-HT6R scaffold and conjugated with tacrine or benzylamine fragments to obtain a new series of MTDLs [72].

Among the studied compounds, derivative **106** (Figure 18) was the most potent compound, having well-balanced activity for the biological targets. Interestingly, compound **106** also inhibited Aβ peptides aggregation in a thioflavine T (ThT) fluorescence assay (94% of inhibition at 10 µM, IC_50_ = 1.27 µM).

**Figure 19:**
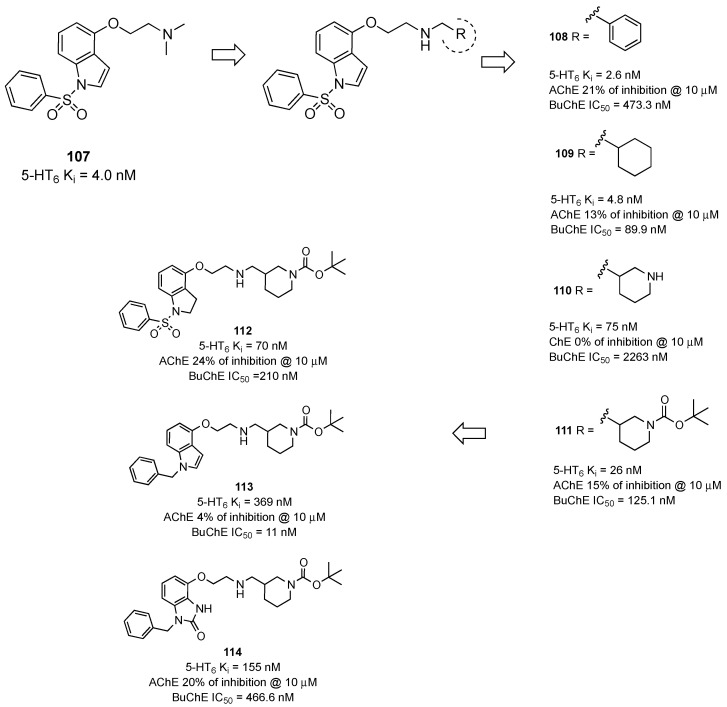
Design and evolution of 4-(2-aminoethoxy)-*N-*(phenylsulfonyl)indole-based MTDLs targeting 5-HT6Rs and BChE [75,77].

The potent 5-HT6R antagonist 4-(2-aminoethoxy)-*N-*(phenylsulfonyl)indole (compound **107**, Figure 19) [74] has been merged with the donepezil-derived fragment benzylamine to obtain a new series MTDLs able to target 5-HT6R, AChE, and BChE [75].

Considering that BChE is overexpressed during the course of AD and plays a rather prominent role over AChE [76], it was investigated if the merging approach could deliver MTDLs able to target 5-HT6R, BChE, and β-amyloid aggregation. Among the studied compounds, derivative **108** (Figure 19) combined 5-HT6R affinity and BChE inhibitory activity and was nearly inactive at AChE. To improve the potency at BChE, compound **108** was further structurally modified by replacing the phenyl ring with a cyclohexyl ring (compound **109**, Figure 19). The cyclohexylmethanamine derivative **109** displayed balanced potency for the biological targets and good inhibitory activity against Aβ aggregation (53% at 10 μM), along with good metabolic stability, a notable safety profile, and the ability to permeate the BBB in mice. The improved potency at BChE of compound **109** was explained by considering the higher basicity compared to compound **108**. Actually, the protonated nitrogen forms crucial interactions at both the 5-HT6R and BChE catalytic site. The cyclohexyl ring was replaced with a piperidine ring (compound **110**, Figure 19) to introduce a second protonation center in order to confirm this hypothesis. This structural modification was detrimental to the activity at BChE. When the piperidine nitrogen was functionalized with a *t*-butyloxycarbonyl (compound **111**, Figure 19), thus abolishing the basicity of the piperidine nitrogen, the inhibitory activity at BChE was recovered. Further modifications of the 1-(phenylsulfonyl)-1*H*-indole fragment of compound **111** provided compounds **112–114** (Figure 19). None of the compounds showed an improved pharmacological profile as compared to **111**. When the 1-(phenylsulfonyl)-1*H*-indole, 1-(phenylsulfonyl)indoline, and 1-benzyl-1,3-dihydro-2H-benzo[*d*]imidazole-2-one fragments of compounds **112–114** were linked to the 1-benzylpiperidine moiety (compounds **115–117**, Table 8), similar results were obtained [77]. In particular, the 1-(phenylsulfonyl)-1*H*-indole fragment (compound **115**) ensured the highest affinity for 5-HT6R (Ki = 22 nM) and BChE inhibitory potency (IC_50_ = 16 nM), most likely because of the higher stability of the interactions established within the catalytic active site of the enzyme. Compound **115** displayed good stability in both human and mouse liver microsomes, safety margins, and free radical scavenging activity, which is helpful for the prevention of cell damage.

**Figure 20:**
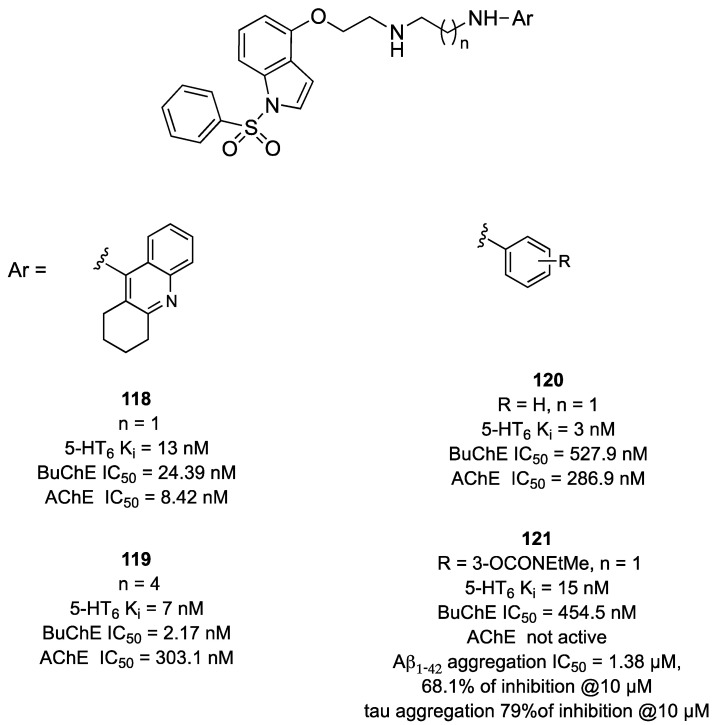
2-[[1-(Phenylsulfonyl)-1*H*-indol-4-yl]oxy]ethan-1-amine derivatives targeting 5-HT_6_R, AChE, and BChE [78].

In a subsequent study, compound **107** (Figure 19) was linked with tacrine, *N-*benzylamine, and phenyl *N-*ethyl-*N-*methylcarbamate, a fragment derived from rivastigmine (Figure 20) [78]. Alkyl linkers of different lengths were included because it was known that increasing the conformational flexibility of the linker would allow cholinesterase-targeting fragments to reach the bottom of the active site gorge and to be better positioned within the site to establish the crucial interactions. Tacrine derivatives showed the highest cholinesterase inhibitory potencies compared to the *N-*benzylamine derivatives (compound **118** vs. compound **120**, Figure 20). Compound **118** was safe despite the tacrine moiety, with acceptable stability but poor predicted capacity to cross the BBB. On the other hand, the introduction of a phenyl *N-*ethyl-*N-*methylcarbamate moiety (compound **121**, Figure 20) yielded pseudo-irreversible inhibition resulting from the carbamoylation of the serine residue in the catalytic site. Compound **121** displayed higher potency than rivastigmine against BChE (IC_50_ = 455 and 2195 nM, respectively). Furthermore, when tested for the ability to inhibit Aβ aggregation, tacrine derivatives were more potent (>60% of inhibition at 10 μM) than the benzylamine counterparts (<60% of inhibition at 10 μM). However, the introduction of substituents on the aromatic ring of the *N-*benzylamine moiety enhanced the inhibitory potency against both Aβ1-42 and tau aggregation. Compound **121** displayed the most promising anti-aggregation properties at 10 μM, with 68% and 79% inhibition of Aβ and tau aggregation, respectively.

**Figure 21:**
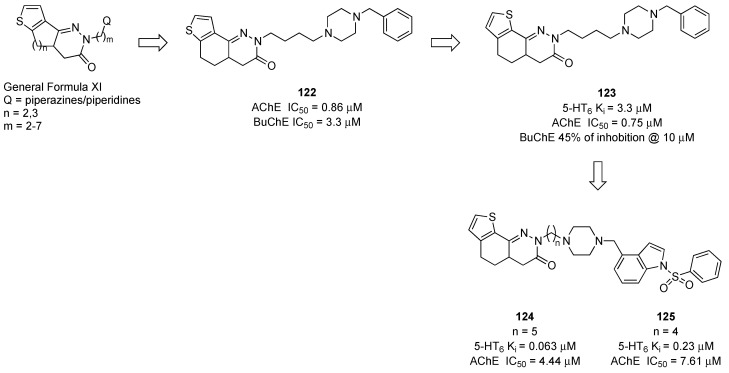
Design strategy to pursue MTDLs targeting 5-HT6R and AChE bearing a thienocycloalkylpyridazinones core [79].

Asproni and colleagues designed novel MTDLs targeting 5-HT6R and AChE by linking the *N-*1-(phenylsulfonyl)-4-(piperazin-1-ylmethyl)-1*H*-indole scaffold (compound **95**, Figure 17) to a series of thienocycloalkylpyridazinones endowed with inhibitory activity at AChE through alkyl chains of variable length (Figure 21). Previous studies evidenced that thienocycloalkylpyridazinones of General Formula XI (Figure 21) can provide compounds displaying AChE inhibition with a wide range of potencies depending on the proper combination of linker/amine moiety. Compound **122** (Figure 21), incorporating the thieno[2,3-*h*]cinnolinone core, showed a high affinity for AChE with a low affinity for BChE. Among the structural modifications performed on compound **122**, the shifting of the sulfur atom from position 7 to 9 on the tricyclic core improved the selectivity toward BChE (compound **123**, Figure 21). The replacement of the benzyl linked to the piperazine with the phenylsulfonyl indole moiety significantly improved the affinity for 5HT6Rs and selectivity over other serotoninergic receptors with a slight reduction of inhibitory activity at AChE (compound **124**, Figure 21). The compounds were predicted to have favorable physicochemical properties for in vivo use, but no data on in vivo activity were reported [79].

**Figure 22:**
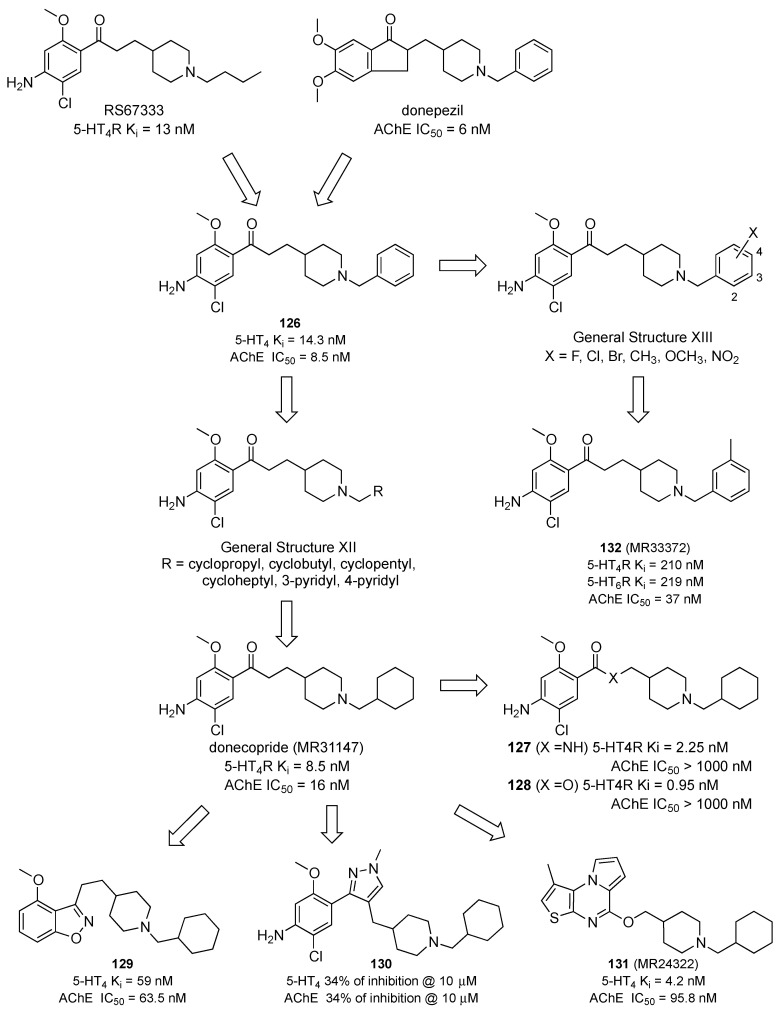
Design strategies and evolution of MTDLs with activity at 5-HT4Rs and AChE [81,82,83].

The observations that the concomitant activation of 5-HT4R and that the inhibition of AChE by administering subactive doses of the 5-HT4R agonist RS67333 and donepezil has synergistic effects on memory performances in mice prompted the group of Dellamagne at the University of Caen to develop new MTDLs combining such activities [80]. Efforts in this direction started by hybridizing the structure of RS67333 with that of donepezil. Replacing the n-butyl group in RS67333 with the benzyl moiety led to compound **126**, which showed balanced activity at both targets (Figure 22). Next, the authors replaced the benzyl group with the saturated counterpart and evaluated lower and higher cyclo homologs (General Structure XII, Figure 22). Among these compounds, donecopride (also known as MR31147, Figure 22) emerged as a balanced 5-HT4R agonist/AChE inhibitor [81]. Donecopride promoted the non-amyloidogenic processing of APP, according to its 5-HT4R agonist properties, leading to preferential release of soluble APPα (sAPPa EC50 = 11.3 nM) and decreased formation of Aβ1-42, in a more efficient fashion than RS67333 (EC_50_ = 27.2 nM) [81].

Subsequent SAR studies on the donecopride structure evidence the importance of the ketone function for AChE activity as the isosteric replacement with an amide or ester function (compounds **127** and **128**, respectively, Figure 22) abolished the AChE inhibitory activity without affecting 5-HT4 affinity. The bioisosteric replacement of the ketone function of donecopride with a benzisoxazole was tolerated (compound **129**, Figure 22), while replacing it with a pyrazole (compound **130**, Figure 22) led to inactive compounds for both targets. Similar results were obtained for a series of pyrrolothienopyrazines exemplified by compound **131** (MR24322, Figure 22) [82].

In subsequent efforts, the same authors modified the structure of compound **126** by introducing substituents on the benzyl ring that might also provide activity at 5-HT6Rs (General Structure XIII, Figure 22). To this end, substituents such as fluoro, chloro, bromo, methyl, methoxy, and nitro were evaluated. Compound **132** (MR33372, Figure 22) emerged, encompassing balanced activity at 5-HT4Rs, 5-HT6Rs, and AChE [83].

## 3. Conclusions

In conclusion, in this review, we have provided an overview of multifunctional ligands that target one or more serotonin receptors and at least one additional target phenotypically linked to a particular disease. Atypical antipsychotic drugs might be considered among the first efforts in this direction as they feature a 5-HT2AR/dopamine D2 receptor antagonist profile. Since the introduction of risperidone, many other drugs targeting 5-HT2ARs, dopamine D2 receptors, and other disease-related targets have been introduced to the market [84]. Notable examples are cariprazine, brexpiprazole, lumateperone, and pivamanserine, which present a composite array of activities at 5-HT1ARs, 5-HT2ARs, 5-HT2BRs, dopamine D3 and D2 receptors, and SERT. These compounds are believed to confer different characteristics in term of efficacy. Future antipsychotic medications will probably include combinations of activities never exploited beyond the combination of activities at serotonin and dopamine receptors. One recent example comes from studies with the compound SEP-363856; its agonist activity at the trace amine-associate receptor, TAAR1, and 5-HT1AR1 has been proposed as a novel strategy to obtain antipsychotic drugs [85]. Other MTDLs might be inspired by real-life use of the combination of the opioid antagonist samidorphan and olanzapine to mitigate olanzapine-induced weight gain [86]. Other combinations of biological activities appear on the horizon as possible strategies, including 5-HT1AR/cannabinoid CB2 and 5-HT1AR/mGlu4R [87,88].

In recent years, the design of MTDLs to treat AD has engaged many research groups worldwide, as AD represents a formidable global health challenge [89]. Available AD medications provide only symptomatic relief; therefore, the scientific community looks for actual disease-modifying therapies. Current efforts to discover AD therapeutics also target different molecular mechanisms and phenomena underpinning the disease. Unsurprisingly, many efforts have been dedicated to pairing AChE inhibition with activity at serotonin receptors involved in cognitive processes, such as 5-HT4Rs and 5-HT6Rs, with donecopride spearheading this field. Clinical trials will undoubtedly tell if donecopride can hold the promise of a disease-modifying therapy [90].

Overall, the design of MTLD ligands for serotonin receptors appears challenging yet feasible, as it has already delivered successful drugs such as atypical antipsychotics. On the other hand, the lesson learned from the massive research efforts made by big pharma in the early 2000s pursuing the combination of 5-HT1AR antagonism/partial agonism with SERT as a novel fast-acting antidepressant deserves some reflection. Merck succeeded in bringing vilazodone to the market as the first drug in its class. However, while vilazodone may have some advantages for the treatment of anxiety in major depressive disorder, it does not appear to be an alternative to SSRIs or other antidepressant therapies [91]. Thus, beyond a convincing rationale and positive results from preclinical studies, the advantage of hitting multiple targets seems to suffer the same translational limitations as magic bullets (i.e., drugs that hit one biological target). Nonetheless, the design of MTDLs with an unexplored combination of activities still represents a mine for discovering new drug candidates.

## Figures and Tables

**Table 1:** Binding affinities for serotonin, dopamine, and sigma receptors of antipsychotic drugs *^a^*.

Name	Structural Formula	5-HT1AR *K*_i_ [nM]	5-HT2AR *K*_i_ [nM]	5-HT7R *K*_i_ [nM]	D2R *K*_i_ [nM]	Sigma 1	Sigma 2
Chlorpromazine	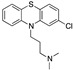	3115	3.2	21	4.8	--	--
Haloperidol	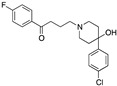	1202	46	378		3.7	48.7
Trifluperazine	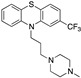	12,022	8.8	288.4	3.8	350 *^b^*	--
Pimozide	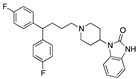	650	19	0.5	0.33	508 *^b^*	--
Risperidone	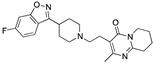	427	0.64	3.5	4.9	950	--
Olanzapine	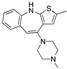	2063	9.2	157	21	5000	--
Aripiprazole	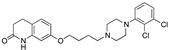	5.6	15.0	10.0	3.3	--	--

*^a^* Data retrieved from the PDSP Ki Database (https://pdsp.unc.edu/databases/pdsp.php; accessed on 15 July 2024); *^b^* non-selective sigma binding affinity.

**Table 2:** Biological data of compounds **18–24** developed by Eli Lilly [32].

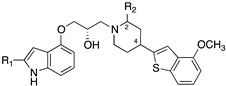							
Compd	R_1_	R_2_	Piperidine Chirality	5-HT1AR *K*_i_ [nM]	SERT *K*_i_ [nM]	% Displacement of the Specific Radioligand Ex Vivo	
						5-HT1AR ^1^	SERT ^2^
**18**	H	H	N.A. ^3^	1.89	12.63	96	0
**19**	CH_3_	H	N.A.	4.83	51.16	N.D. ^4^	N.D.
**20**	H	CH_3_	2*R*,4*R*	2.76	14.71	72	30
**21**	H	CH_3_	2*S*,4*S*	14.45	13.59	60	12
**22**	H	CH_3_	2*S*,4*R*	3.64	0.27	87	89
**23**	H	CH_3_	2*R*,4*S*	8.47	1.15	67	92
**24**	CH_3_	CH_3_	2*S*,4*R*	14.35	0.24	100	74

^1^ Inhibition of ex vivo binding of [^3^H]-8-OH-DPAT (1 nM) in the frontal cortex homogenates at 30 mg/kg po; ^2^ Inhibition of ex vivo binding of [^3^H]-paroxetine (0.1 nM) in the frontal cortex homogenates at 30 mg/kg po; ^3^ Not applicable; ^4^ Not determined.

**Table 3:** Biological data of compounds **58**–**66** developed by GSK [43].

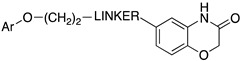					
Compd	Ar	LINKER	5-HT1AR p*K*_i_	IA ^1^	SERT p*K*_i_
**58**	4-indolyl	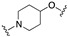	8.4	0.2	8.3
**59**	1-naphthyl	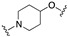	8.6	0.4	7.0
**60**	1-isoquinolinyl	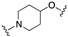	8.1	0.8	7.3
**61**	4-quinolinyl	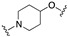	7.0	N.D.^2^	7.0
**62**	8-quinolinyl	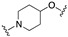	8.2	0.5	7.4
**63**	5-quinolinyl	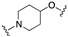	7.9	0.1	7.5
**64**	5-quinolinyl	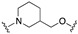	8.8	0.3	7.1
**65**	5-quinolinyl	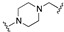	8.0	0.2	7.5
**66**	5-quinolinyl	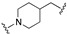	8.9	0.2	8.2

^1^ 5-HT1AR intrinsic activity expressed relative to the 5-HT response; ^2^ Not determined.

**Table 4:** Biological data of compounds **67–75** developed by GSK [44].

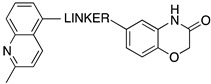					
Compd	LINKER	5-HT1AR p*K*_i_	5-HT1BR p*K*_i_	5-HT1DR p*K*_i_	SERT p*K*_i_
**67 (SB-6499115)**	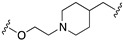	8.6	8.0	8.8	8.1
**68**	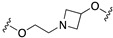	7.3	7.3	8.1	7.2
**69**	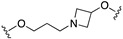	7.1	6.5	6.8	N.D.
**70**	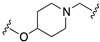	<5.0	<5.6	5.0	6.7
**71**	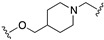	<5.8	<5.0	5.9	7.5
**72**	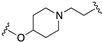	6.5	7.6	8.2	7.0
**73**	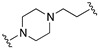	9.6	9.3	9.7	8.4
**74**	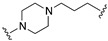	8.6	7.3	8.0	7.6
**75**	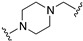	6.2	<5.0	6.9	6.1

**Table 5:** 4-Arylpiperazine and 4-arylpiperidine derivatives with mixed sigma and 5-HT1AR/5-HT2AR affinity [66].

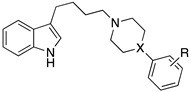						
Compd	X	R	Sigma 1	Sigma 2	5-HT1AR *K*_i_ [nM]	5-HT2AR *K*_i_ [nM]
**86**	N	2-OCH_3_	14	21	17	150
**87**	CH	2-OCH_3_	4.5	3.3	56	83
**88**	N	4-F	5.6	1.3	37	14
**89**	CH	H	1.5	0.48	110	25
**90**	CH	4-F	1.4	4.0	27	34

**Table 6:** Compounds with affinity for sigma receptors and 5-HTRs [67].

Receptor Affinity (p*K*_i_)	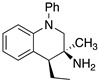 (±)-91	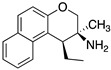 (±)-92	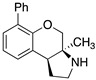 (±)-93
5-HT1AR	7.13	6.5	6.7
5-HT2BR	6.1	7.7	6.8
5-HT7R	6.0	6.18	7.63
Sigma 1	6.5	6.0	7.4
Sigma 2	6.2	5.7	6.9

**Table 7:** 4-(Piperazin-1-yl)-1*H*-indoles targeting 5-HT6R, AChE, and BChE.

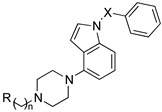						
Compd	R	N	X	5-HT6R *K*_i_ [nM]	AChE IC_50_ [nM]	BChE IC_50_ [nM]
**96**	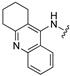	2	SO_2_	10	43.1	16.8
**97**		6	SO_2_	2.0	12.9	8.2
**98**		2	CH_2_	36	26	5
**99**		6	CH_2_	94	13	15
**100**		8	SO_2_	130	1.3	12.4
**101**	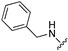	6	SO_2_	2.0	(37%)	2384
**102**		6	CH_2_	510	8217	1562
**103**	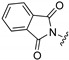	2	CH_2_	17	(<10%)	6820
**104**		3	CH_2_	149.8	(<10%)	3440

**Table 8:** Biological profile of compounds **115–117** [77].

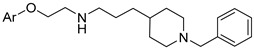				
Compd	Ar	5-HT_6_ *K*_i_ [nM]	AChE IC_50_ [nM]	BChE IC_50_ [nM]
**115**	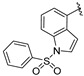	22	930	16
**116**	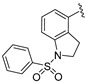	598	821	487
**117**	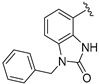	480	544	613

## Data Availability

No new data were created in this work.
